# Supplementary material for: The Utility of Blood Cultures in Non-Febrile Patients and Patients with Antibiotics Therapy in Internal Medicine Departments
Source: J Clin Med. 2025 Mar 30;14(7):2373. doi: 10.3390/jcm14072373 (PMC11989235; doi:10.3390/jcm14072373)
Supplement: Supplementary file 1 [file jcm-14-02373-s001.zip › jcm-3331971-supplementary.pdf]

Table S1: Association between the bacteriological yield and patients' charactierstics

Characteristics	True bacteremia, n = 4,045(5.5)	Contamination and no growth, n=69,745(94.5%)	SMD
Optimal timing	1,562(38.6%)	24,054(34.5%)	0.086
Age(y.)	66.13±17.41	65.25±18.44	0.049
Male, n (%)	2,454(60.7%)	40,950(58.7%)	
Comorbidities			
CHF, n (%)	1,047(25.9%)	15,621(22.4%)	0.082
Stroke, n (%)	1,020(25.2%)	17,172(24.6%)	0.014
Dementia, n (%)	308(7.6%)	4557(6.5%)	0.042
Chronic pulmonary disease, n (%)	1,111(27.5%)	18,611(26.7%)	0.018
Rheumatological disease, n (%)	135(3.3%)	2,723(3.9%)	0.030
Diabetes mellitus, n (%)	1,740(43.0%)	27,124(38.9%)	0.084
Diabetes mellitus with chronic complications, n (%)	684(16.9%)	10,177(14.6%)	0.064
Peripheral Vascular disease, n (%)	728(18.0%)	10,632(15.2%)	0.074
Malignancy, n (%)	835(20.6%)	13,298(19.1%)	0.040
Renal disease, n (%)	1,285(31.8%)	18,847(27.0%)	0.104
Vital signs			
Pulse (bpm)	99.0±39.6	94.1±21.8	0.154
Systolic Blood pressure(mmHg)	127.3±23.8	133.2±22.7	0.258
Blood tests			
White blood cells (mm^3/mL)	11.83±8.75	11.8±8.7	0.240
C-reactive protein (mg/dL)	15.29±13.78	12.72±11.19	0.239
